# Association between Age at Diagnosis of Type 2 Diabetes and Subsequent Risk of Dementia and Its Major Subtypes

**DOI:** 10.3390/jcm13154386

**Published:** 2024-07-26

**Authors:** Da Hea Seo, Mina Kim, Yongin Cho, Seong Hee Ahn, Seongbin Hong, So Hun Kim

**Affiliations:** 1 Division of Endocrinology and Metabolism, Department of Internal Medicine, Inha University College of Medicine, Incheon 22332, Republic of Korea; dahea@inha.ac.kr (D.H.S.); choyorin@gmail.com (Y.C.);; 2 Department of Data Science, Hanmi Pharmaceutical Company Limited, Seoul 05545, Republic of Korea; mina.kim92@hanmi.co.kr

**Keywords:** type 2 diabetes mellitus, dementia, age of diabetes onset

## Abstract

**Background/Objectives**: Type 2 diabetes mellitus (T2DM) is a major contributor to cognitive decline and dementia in older adults; however, the role of the age of onset of T2DM in younger patients remains uncertain. We explored the association between the risk of dementia and its subtypes in relation to the age at T2DM diagnosis. **Methods:** This population cohort study included a total of 612,201 newly diagnosed T2DM patients. The controls were randomly selected from the general population and matched at a 1:2 ratio based on the propensity score. The outcomes of interest were all-cause dementia, Alzheimer’s disease (AD), and vascular dementia (VD). The association of T2DM with dementia was stratified by the age at diagnosis of T2DM. **Results**: The mean ages of the subjects in the T2DM and control groups were 55.7 ± 13.0 and 55.7 ± 13.0. The patients with T2DM diagnosed at <50 years had the highest excess risk for most outcomes relative to the controls, with a hazard ratio (HR) (95% CI) of 3.29 (3.11–3.49) for all-cause dementia, 4.08 (3.18–5.24) for AD, and 5.82 (3.84–8.81) for VD. All risks were attenuated progressively with each increasing decade at the diagnostic age, but remained significant; for T2DM diagnosed at ≥80 years, the HR (95% CI) was 1.38 (1.34–1.41) for all-cause dementia, 1.35 (1.31–1.40) for AD, and 1.98 (1.70–2.30) for VD. **Conclusions**: We need to stratify T2DM management according to the age of diagnosis. Physicians should closely monitor cognitive function in patients with T2DM, especially in younger individuals.

## 1. Introduction

The prevalence of diabetes continues to rise worldwide [1]. Type 2 diabetes mellitus (T2DM) has conventionally been considered a disease of middle and older age; however, the rapid growth of T2DM has been observed among younger adults, now constituting between 15 and 20% of all adults with T2DM worldwide [2]. Recent evidence indicates an accelerated disease course and a subsequent higher risk of cardiovascular morbidity and mortality in patients with early-onset T2DM than in those with late-onset T2DM [3].

Dementia represents a group of diseases that are associated with a loss of memory, cognitive function, and a subsequent decline in the activities of daily living, which place social and economic burdens on patients, their families, and the government [4]. T2DM is recognized as a risk factor for cognitive decline and dementia [5]. Evidence from meta-analyses of prospective studies suggests that individuals with T2DM have a higher risk of Alzheimer’s disease (AD) and vascular dementia (VD) than individuals without T2DM [6,7]. Although the mechanisms involved in the association between T2DM and dementia risk remain unknown, perturbed brain glucose metabolism due to insulin resistance has been recognized as one of the main drivers of the development of incident AD and other dementias [8]. Moreover, synaptic dysfunction, microglial overactivation, mitochondrial dysfunction, and neuronal apoptosis contribute to the pathogenesis of dementia in patients with T2DM [8]. However, studies that have specifically examined the age at onset of diabetes or the duration of diabetes are limited, as studies on dementia usually include individuals older than 65 years. A recent population-based study in the UK reported that a younger age at the diagnosis of diabetes is significantly associated with a higher risk of all-cause dementia, suggesting a significant association between the age at T2DM diagnosis and dementia risk [9]. However, it was unable to distinguish dementia subtypes, and the majority of the study participants were white. Furthermore, no previous studies have compared the dementia risk with the age at T2DM diagnosis.

Given the increasing prevalence of early-onset T2DM, especially in Asian countries, we explored the association between the age at onset of T2DM and the risk of dementia and its subtypes relative to matched controls, using the National Health Insurance Service database. We also examined the association between glycemic control and the risk of dementia in patients with T2DM.

## 2. Materials and Methods

### 2.1. Design, Study Setting, and Participants

The National Health Insurance Service (NHIS) is a government-operated health insurance program that covers almost the entire Korean population. The National Health Information Database (NHID) of the NHIS consists of comprehensive sets of health information. This study’s protocol was approved by the IRB of Inha University Hospital (2023-01-003), and the requirement for informed consent was waived because pseudonymized data were analyzed.

After excluding patients who were prescribed anti-diabetic drugs or had at least 1 recorded diagnosis of T2DM (International Classification of Diseases [ICD]-10 codes E11–14) during the previous 24 months, a total of 635,557 individuals aged ≥18 years with a diagnosis of T2DM between 1 January 2012 and 31 December 2014 were selected. New-onset T2DM was regarded as the presence of at least one recorded diagnosis of T2DM, with accompanying prescription codes for any anti-diabetic drugs (index date). Patients with missing covariate information (*n* = 949) and those diagnosed with dementia before the index date (*n* = 22,149) were excluded.

**Figure 1:**
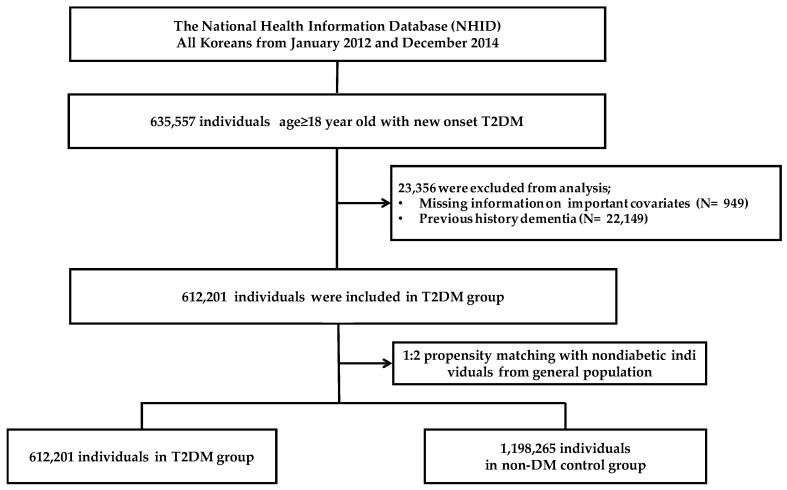
Flowchart for study design.

For the matched controls, the index date was randomly selected based on the distribution of the index date for the T2DM group, after excluding those with at least 1 recorded diagnosis of T2DM (ICD-10 codes E11-14) or dementia (F00, F01, F02, F03, G30, or G31), a prescription for anti-diabetic drugs, or a fasting glucose level ≥ 126 mg/dL during health checkups provided by the NHIS between 1 January 2012 and 31 December 2014. Propensity score matching was then performed to reduce the imbalance in the distribution of the baseline covariates between the two groups using the 1:2 greedy nearest neighbor algorithm. The following covariates were included in the propensity score: age, sex, index date, and history of cardiovascular disease (CVD) within one year of the index date. The presence of CVD was defined as previous diagnoses with the corresponding ICD-10 codes, procedure codes, and treatment codes within one year of the index date (Table S1) [10]. Severe hypoglycemia was defined as hospitalization or a visit to the emergency department with a primary diagnosis of hypoglycemia and the corresponding ICD-10 codes during the study period (Table S1). Finally, a total of 1,810,466 participants, including 612,201 patients with T2DM and 1,198,265 controls, were included in the final analysis (Figure 1).

Among this database, a total of 1,235,416 participants (391,110 individuals from the T2DM group and 844,306 individuals from the control group) completed health checkups provided by the NHIS between 1 January 2012 and 31 December 2014 and were included in the subgroup analysis. The association between T2DM, dementia outcomes by age at the diagnosis of T2DM and glycemic control was examined after adjusting for multiple clinical factors.

### 2.2. Covariates

Covariate information was obtained from the National Health Screening Examination data collected between 2012 and 2014. Comorbidities (hypertension, dyslipidemia, and chronic kidney disease) were defined as previously described [11]. Household income information was obtained from a self-administered questionnaire and divided into quartiles. The participants’ smoking status, alcohol consumption, and physical activity were assessed using questionnaires. The participants were categorized as non-smokers, ex-smokers, or current smokers. Heavy drinkers were defined as those with alcohol consumption > 30 g/day [12].

### 2.3. Outcome Measures and Follow-Up

Dementia was determined based on ICD-10 codes (F00, F01, F02, F03, G30, or G31) using medical expense claims that had been submitted to the NHIS through an outpatient clinic more than three times or for at least one hospital admission [11]. AD was diagnosed using the F00 and G30 codes; VD using the F01 code; and other dementias using the F02, F03, and G31 codes. When two or more dementia diagnosis codes were registered together, the dementia subtype was determined according to the primary diagnosis. Each participant was followed from the index date until the earliest occurrence of any study outcome, death, or the end of the study period (31 December 2019).

### 2.4. Statistical Analysis

Continuous and categorical variables were presented as means (standard deviations) and numbers (percentages), respectively. The propensity score was estimated using the multiple logistic regression model of the baseline covariates for new-onset T2DM. The covariate balance was calculated as the standardized differences before and after propensity score matching. Significant imbalances were defined as standardized differences ≥ 10% [13]. All occurrences of each outcome were included. A Cox proportional hazards regression model in propensity score-matched pairs was used to estimate the hazard ratios (HR) for the study outcomes, with adjustment for the occurrence of severe hypoglycemia. The proportional hazards assumption for each model was tested using Schoenfeld residuals. Each model was validated based on the proportional hazards assumption; therefore, the Cox proportional hazards regression model was fitted to these data. Participants were classified into five groups according to their age at onset of T2M, as follows: <50 years, 50–59 years, 60–69 years, 70–79 years, and ≥80 years. Analyses of all outcomes were repeated for each decade of age at diagnosis of T2DM to examine the associations between age at diagnosis of T2DM and dementia and its subtypes. The same analyses were repeated in a subgroup of participants who underwent health checkups provided by the NHIS, after adjusting for sex, smoking, drinking, physical activity, income, BMI, systolic blood pressure (SBP), total cholesterol, triglyceride (TG), fasting blood glucose (FBG), history of CVD, and occurrence of severe hypoglycemia during the follow-up period. To investigate whether the association between T2DM and dementia outcomes differs by glycemic control, we conducted Cox proportional hazards regression analyses according to categories of FBG concentration (<95, 95–124, 125–139, 140–199, and ≥200 mg/dL) [14] in patients with T2DM. Statistical analyses were performed using the SAS software (version 9.4; SAS Institute, Cary, NC, USA). A *p*-value < 0.05 was considered to be statistically significant.

## 3. Results

### 3.1. Baseline Characteristics

A total of 612,201 participants diagnosed with new-onset T2DM between 1 January 2012 and 31 December 2014 were included. The baseline characteristics of the patients are shown in Table 1. Those with T2DM had a higher prevalence of angina pectoris and peripheral artery disease than the control group. Following propensity matching, the baseline characteristics were well-balanced between the groups and the cohort included 1,810,466 participants: 612,201 participants in the T2DM group and 1,198,265 participants in the control group (Figure 1). The mean ages of the T2DM and control groups were 55.7 ± 13.0 (range: 18–113) years and 55.7 ± 13.0 (range: 18–102) years, and 60% of the subjects were male.

### 3.2. Dementia Outcomes

After 6.3 years of follow-up, dementia occurred in 66,186 (10.8%) patients with T2DM compared to 85,358 (7.1%) control subjects. The dementia outcomes over time for patients with T2DM versus the controls are presented in Table 2. There was a higher risk of dementia in the T2DM group. Compared with the control subjects without T2DM, patients with T2DM had a 1.6-fold increased risk of all-cause dementia and AD (HR [95% CI]: 1.61 [1.59–1.62] and 1.58 [1.55–1.60], respectively) and a 2.1-fold increased risk of VD (HR 2.11 [1.98–2.25]). The results were generally comparable in both men and women except for VD, with evidence of a stronger effect in men than in women (HR [95% CI]: 2.46 [2.23–2.70] and 1.84 [1.68–2.02] for men and women, respectively).

When the analysis was repeated, stratified by the age at diagnosis of T2DM, there was a higher risk of dementia outcomes regardless of the age at diagnosis of T2DM compared to the control subjects in the same age group, and those with T2DM diagnosed at <50 had the highest excess relative risk of all-cause dementia, AD and VD, as shown in Table 3 (HR 3.29 [3.11–3.49], HR 4.08 [3.18–5.24] and HR 5.82 [3.84–8.81], respectively). The incremental risks declined with each subsequent decade at the time of T2DM diagnosis. For participants with T2DM diagnosed at ≥80 years, the excess relative risk of T2DM compared to the controls regarding dementia outcomes still remained significant (HR 1.38 [1.34–1.41], HR 1.35 [1.31–1.40] and HR 1.98 [1.70–2.30] for all-cause dementia, AD and VD, respectively).

**Figure 2:**
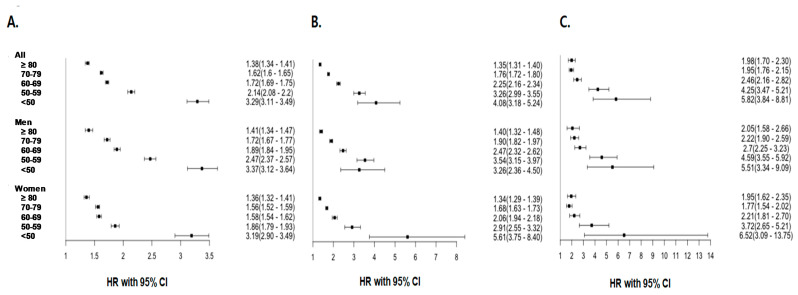
Adjusted hazard ratios (95% confidence interval [CI]) for all dementia (**A**), Alzheimer’s disease (**B**), and vascular dementia (**C**) in patients with type 2 diabetes mellitus according to the age at diagnosis of type 2 diabetes, in comparison with matched controls. The analyses were adjusted for severe hypoglycemia.

When outcomes were examined separately by sex, the results were generally comparable in terms of the patterns observed or the risk, according to the age at diagnosis (Table 3 and Figure 2), although the HRs were usually higher in men for all dementia outcomes except for the group with an age at diagnosis of T2DM < 50 years (*p* < 0.0001 for sex by age interactions for all outcomes). Compared with those without diabetes, women with T2DM diagnosed at age < 50 years showed a 5.6-fold increased risk of the development of AD and a 6.5-fold increased risk of the development of VD; meanwhile, there was a 3.3-fold and 5.5-fold greater risk of AD and VD in men. The higher excess dementia risk in both men and women was attenuated by each subsequent decade added to the age at T2DM diagnosis, but remained significant at age at ≥80 of diagnosis.

### 3.3. Subgroup Analysis in Participants with Health Checkups Provided by the NHIS

Table S2 shows the baseline characteristics of the participants who completed health checkups provided by the NHIS, stratified by dementia status. A total of 1,235,416 participants were included, including 102,244 who developed dementia during the study period. Participants who developed dementia were more likely to be women, older, have a lower BMI, and have a lower physical activity level than those who did not develop dementia. At baseline, a higher prevalence of type 2 diabetes, hypertension, dyslipidemia, cancer, chronic kidney disease and CVD was observed in individuals who developed dementia. When the dementia outcomes were examined in the cohort of patients who underwent health checkups provided by the NHIS and stratified by the age at type 2 diabetes diagnosis, the results were generally comparable in terms of the patterns observed or risk according to the age at diagnosis, even after adjustment for sex, smoking, alcohol consumption, physical activity, income, BMI, SBP, total cholesterol, TG, FBS, history of CVD, and occurrence of severe hypoglycemia (Table 4).

### 3.4. Results Stratified by Glycemic Control

The association between fasting blood glucose (FBG) and the risk of dementia and its subtypes in patients with T2DM was examined using the health checkup data (Table 5). High FBG levels were associated with an increased risk of both AD and VD among those with T2DM, although the magnitude of the association differed according to dementia subtype. The strongest association was observed for VD; compared with patients with a fasting glucose concentration 95–120 mg/dL, those with a fasting glucose concentration ≥ 200 mg/dL had a 47% higher risk of VD (HR 1.47 [1.15–1.89]). These associations were independent of age, sex, income, smoking, alcohol consumption, BMI, diastolic and systolic blood pressure, LDL cholesterol, HDL cholesterol, and CVD history.

## 4. Discussion

To the best of our knowledge, this is the first study to examine the association between the age at T2DM diagnosis and the risk of dementia. Overall, diabetes was associated with a 50–70% increased risk of all-cause dementia and AD in both women and men. Furthermore, individuals with diabetes had an approximately 110% greater risk of developing VD than those without, and there was evidence of a stronger effect in men than in women. Moreover, a younger age at T2DM diagnosis was associated with a higher subsequent risk of all dementia outcomes. The risk of VD was even more markedly elevated in those with T2DM diagnosed at a younger age, where the incremental relative risk was ~5–6 times higher than in the matched controls. The incremental risks associated with T2DM were attenuated by each subsequent decade added to the age at T2DM diagnosis, but remained significant even at age ≥80 for all dementia outcomes. We also provided evidence that in individuals with T2DM, poor glycemic control is associated with an elevated risk of both AD and VD.

Our study confirms previous findings suggesting that T2DM is a risk factor for dementia and that this risk differs by subtype of dementia [7]. A large meta-analysis of 14 prospective studies reported that individuals with T2DM have a 60% higher risk of all-cause dementia and a 40% higher risk of AD than those without T2DM [7]. The risk of VD was even higher in patients with T2DM; men with diabetes had a 140% higher risk of developing VD, while women had an 80% higher risk. However, most studies have not explicitly considered the effect of the age at diabetes diagnosis on the risk of dementia because they usually include participants older than 65 years old. In a recent, prospective cohort study of 10,095 participants, a younger age at the onset of T2DM was significantly associated with a higher risk of incident dementia; at age 70, every 5-year decrease in the age at onset of T2DM was significantly associated with a greater risk of dementia, with an HR (95% CI) of 1.24 (1.06–1.46) [9]. However, the association between T2DM and its subtypes of dementia and sex differences in the T2DM-associated risk of dementia have not yet been investigated. In this nationwide Korean study involving 1,810,466 participants, we demonstrated that a younger age at T2DM diagnosis is associated with a higher subsequent risk of all-cause dementia as well as its subtypes, with the excess risks becoming less evident in those with T2DM diagnosed at an older age. Moreover, the excess relative effect of diabetes on incident VD was greater than AD, and the adverse consequences of diabetes on dementia were stronger in men than in women, confirming recent findings from the ADVANCE trial [15].

The mechanisms involved in the association between diabetes and dementia are unclear, but may be multifactorial. Plausible biological mechanisms include insulin resistance, with the subsequent impairment of cerebral glucose metabolism, hyperglycemic neurotoxicity, chronic inflammatory processes, oxidative stress and microvascular dysfunction [16,17,18,19]. However, the exact mechanism underlying the link between the age at T2DM diagnosis and the risk of dementia remains unclear. In a recent study using the Swedish National Diabetes Registry, the risk factors that accounted for the association with dementia subtypes were investigated. Although the risk factors differed among dementia subtypes, the shared risk factors with the greatest relative influence were age, systolic blood pressure, diabetes duration, existing CVD and BMI [20]. In this study, patients who were diagnosed with T2DM at age <50 years had the highest excess risk of dementia, and almost 32% of the patients were aged <50. Accumulating evidence suggests that early-onset T2DM is associated with a poorer metabolic profile, a higher prevalence of obesity, and a more rapid decline in β-cell function than late-onset T2DM, resulting in the early development of microvascular and macrovascular complications, as well as premature death [21]. Thus, we can speculate that the aggressive phenotypes of those patients contributed to their higher risk of dementia. Moreover, diabetes was considered a strong risk factor for early-onset dementia (age < 65 years), with an HR (95% CI) of 1.65 (1.15–2.36) even after adjusting for apolipoprotein E status, one of the major genetic risk factors in early-onset dementia [22]. Although future research is needed to clarify how different ages at T2DM diagnosis contribute to the development of dementia, the impact of being younger at T2DM diagnosis on the risk of dementia appears to be stronger than that of being older at the time of diagnosis.

Interestingly, older patients with a diagnosis of T2DM at ≥80 still had significant excess risks for both AD and VD relative to their counterparts, different from the results of a recent UK study where late-onset diabetes (~65 years at diagnosis of T2DM) was not significantly associated with subsequent dementia [9]. Similarly, a Swedish Twin Registry study demonstrated that mid-life-onset diabetes (age < 65 years) was associated with a 2.41-fold increased risk of dementia, while no significant association was found in patients with late-onset diabetes (age ≥ 65 years) [23]. Although no direct comparison can be made among different ethnic groups, the differences may be attributed to differences in genetic and unmeasured environmental factors [23]. Moreover, in a recent study comparing the differences in the cardiometabolic risk profiles between Chinese and Finnish older adults with T2DM, Chinese individuals had higher insulin resistance and triglyceride levels than Finnish individuals [24], both of which are significantly associated with dementia [19,25]. Furthermore, differences in body composition, especially visceral fat accumulation, may explain this finding, since Asians tend to have more visceral fat regardless of BMI [26], which is also an important factor contributing to cognitive decline [27]. Nevertheless, our findings indicate that the active surveillance of T2DM is still required for older patients.

Most previous epidemiological studies have mainly investigated the association between dementia and the diagnosis or the presence of diabetes; however, evidence regarding the role of glycemic control in the association between T2DM and the risk of dementia is limited and there are contradictory results [28,29]. In a large-scale cohort study of 457,902 patients with diabetes, a dose–response association between the longitudinal hemoglobin A1c (HbA1c) concentration and the risk of dementia in patients with T2DM was observed; in comparison to the reference group (HbA1c concentration of 6–7%), patients with HbA1c levels of 8–9%, 9–10%, and ≥10% had an increased risk of dementia at rates of 15%, 26%, and 40%, respectively [30]. Similarly, a recent UK study demonstrated a linear association between the HbA1c concentration and risk of dementia in patients with T2DM, but the magnitude of the association differed according to the subtype of dementia; the strongest association was found for VD, with an adjusted HR (95% CI) of 1.93 (1.54–2.43) for a HbA1c level of ≥10.1%, in comparison to a HbA1c level of ≤6.9% [20]. In another prospective, community-based cohort study with 2067 participants, high glucose levels were also related to an increased risk of dementia among those with diabetes; participants with a glucose level of 190 mg/dL had an adjusted HR (95% CI) of 1.40 (1.12–1.76) for dementia, compared to a glucose level of 160 mg/dL [28]. In this study, compared to the reference group (95 ≤ FBG ≤ 124 mg/dL), those with FBG ≥ 200 mg/dL had an increased risk of both AD and VD, even after adjusting various confounding factors. Finally, although a large sample size may uncover weak effects that are of little clinical importance, in this study with large sample sizes, the magnitude of the HR was medium to large, indicating that the results were clinically significant.

This study had some limitations. Our data are derived exclusively from the NHID, comprising almost exclusively Koreans; therefore, further studies in different countries and with more heterogeneous populations are needed. This analysis relied on claims data, which may have been inaccurate compared with the diagnoses obtained from a medical chart, neuroimaging tests, or neuropsychological tests. Despite the 6.3 years of follow-up, patients with early-onset T2DM had not yet reached an age at which dementia was more prevalent. A longer follow-up period would allow the further examination of the association between the age at T2DM onset and the risk of dementia.

Our study has several strengths. First, it was a large national population-based study that included almost all Koreans, and followed the participants from the time they were diagnosed with T2DM to assess the risk of dementia. Second, we included a large proportion of elderly patients aged >80 years who were newly diagnosed with T2DM. To date, no comprehensive study has assessed the risk of T2DM-related dementia and its subtypes according to the age at diagnosis, in comparison with matched controls in an Asian cohort. In addition, we only included subjects with newly diagnosed T2DM to fully adjust for the effects of diabetes duration, which is independently associated with a high risk of dementia [31].

## 5. Conclusions

This large population cohort study suggests that the dementia risk associated with T2DM differs markedly by age at diagnosis, with the highest dementia risks in those with an early T2DM diagnosis. Our data highlight the importance of the close monitoring of cognitive function in patients with diabetes, especially in those with early-onset T2DM.

## Figures and Tables

**Table 1:** Baseline characteristics of type 2 diabetes mellitus and matched controls at diagnosis.

	Before Matching			After Matching		
	T2DM	Control	SMD	T2DM	Control	SMD
N	612,201	1,763,724		612,201	1,198,265	
Age (years)	55.7 ± 13.0	55.9 ± 13.3	0.0282	55.7 ± 13.0	55.7 ± 13.0	−0.0004
Men	367,081 (60.0)	1,048,921 (59.5)	0.0100	367,081 (60.0)	723,487 (60.4)	−0.0085
Index year						
2012	251,606	712,771	0.0140	251,606	491,982	0.0008
2013	201,550	583,031	−0.0029	201,550	395,541	−0.0019
2014	159,045	467,922	−0.0125	159,045	310,742	0.0011
History of CVD						
Myocardial infarction	3528 (0.6)	5485 (0.3)	0.0399	3528 (0.6)	4015 (0.3)	0.0358
CABG	28 (0.005)	50 (0.003)	0.0029	28 (0.005)	44 (0.004)	0.0014
PCI with stent	1064 (0.2)	1171 (0.1)	0.0310	1064 (0.2)	1028 (0.1)	0.0244
Unstable angina	6458 (1.1)	9291 (0.5)	0.0596	6458 (1.1)	8009 (0.7)	0.0418
Angina pectoris	25,324 (4.1)	37,858 (2.1)	0.1143	25,324 (4.1)	32,054 (2.7)	0.0806
Atrial fibrillation	5674 (0.9)	10,012 (0.6)	0.0417	5674 (0.9)	8059 (0.7)	0.0286
Heart failure	10,253 (1.7)	14,209 (0.8)	0.0786	10,253 (1.7)	10,745 (0.9)	0.0691
Stroke	17,534 (2.9)	42,075 (2.4)	0.0299	17,534 (2.9)	28,475 (2.4)	0.0305
Hemorrhagic stroke	1993 (0.3)	6225 (0.4)	−0.0047	1993 (0.3)	3998 (0.3)	−0.0014
Ischemic stroke	13,777 (2.3)	32,987 (1.9)	0.0268	13,777 (2.3)	21,790 (1.8)	0.0306
PAD	51,552 (8.4)	92,223 (5.2)	0.1268	51,552 (8.4)	78,155 (6.5)	0.0722

Values are expressed as mean ± standard deviation or number (%). CVD, cardiovascular disease; CABG, coronary artery bypass graft; PAD, peripheral artery disease; PCI, percutaneous coronary intervention; SMD, standardized mean difference; T2DM, type 2 diabetes mellitus.

**Table 2:** Event rates and hazard ratios for all dementia subtypes and their subtypes.

	No of Events	IR (1000 PY)		HR (95% CI) *	*p*	HR (95% CI) *		*p*
All								
All dementia								
Control	85,358	11.25		1.00 (Reference)		1.00 (Reference)		
T2DM	66,186	17.99		1.61 (1.59–1.63)	<0.0001	1.61 (1.59–1.62)		<0.0001
Alzheimer disease								
Control	32,135	4.36		1.00 (Reference)		1.00 (Reference)		
T2DM	24,221	6.88		1.58 (1.56–1.61)	<0.0001	1.58 (1.55–1.60)		<0.0001
Vascular dementia								
Control	1802	0.25		1.00 (Reference)		1.00 (Reference)		
T2DM	1806	0.52		2.11 (1.98–2.26)	<0.0001	2.11 (1.98–2.25)		<0.0001
Men								
All dementia								
Control	32,249	6.98		1.00 (Reference)		1.00 (Reference)		
T2DM	27,502	12.37		1.78 (1.75–1.81)	<0.0001	1.78 (1.75–1.81)		<0.0001
Alzheimer disease								
Control	10,818	2.39		1.00 (Reference)		1.00 (Reference)		
T2DM	9073	4.21		1.77 (1.72–1.82)	<0.0001	1.76 (1.71–1.81)		<0.0001
Vascular dementia								
Control	784	0.17		1.00 (Reference)		1.00 (Reference)		
T2DM	913	0.43		2.46 (2.24–2.71)	<0.0001	2.46 (2.23–2.70)		<0.0001
Women								
All dementia								
Control	53,109	17.88		1.00 (Reference)		1.00 (Reference)		
T2DM	38,684	26.59		1.50 (1.48–1.52)	<0.0001	1.50 (1.48–1.51)		<0.0001
Alzheimer disease								
Control	21,317	7.51		1.00 (Reference)		1.00 (Reference)		
T2DM	15,148	11.10		1.48 (1.45–1.51)	<0.0001	1.48 (1.45–1.51)		<0.0001
Vascular dementia								
Control	1018	0.37		1.00 (Reference)		1.00 (Reference)		
T2DM	893	0.68		1.84 (1.69–2.02)	<0.0001	1.84 (1.68–2.02)		<0.0001

* Adjusted for severe hypoglycemia. CI, confidence interval; IR, incidence rate; HR, hazard ratio; T2DM, type 2 diabetes mellitus.

**Table 3:** Hazard ratios for all dementia and its subtypes, stratified by age for type 2 diabetes mellitus diagnosis and sex.

	All Dementia		Alzheimer Disease		Vascular Dementia	
	HR (95% CI)	*p* Value	HR (95% CI)	*p* Value	HR (95% CI)	*p* Value
All						
≥80	1.38 (1.34–1.41)	<0.0001	1.35 (1.31–1.40)	<0.0001	1.98 (1.70–2.30)	<0.0001
70–79	1.62 (1.59–1.65)	<0.0001	1.76 (1.72–1.80)	<0.0001	1.95 (1.76–2.15)	<0.0001
60–69	1.72 (1.68–1.75)	<0.0001	2.25 (2.16–2.34)	<0.0001	2.46 (2.16–2.82)	<0.0001
50–59	2.14 (2.08–2.20)	<0.0001	3.26 (2.99–3.55)	<0.0001	4.25 (3.47–5.21)	<0.0001
<50	3.29 (3.11–3.49)	<0.0001	4.08 (3.18–5.24)	<0.0001	5.82 (3.84–8.81)	<0.0001
Men						
≥80	1.40 (1.34–1.47)	<0.0001	1.40 (1.32–1.48)	<0.0001	2.05 (1.58–2.66)	<0.0001
70–79	1.72 (1.67–1.77)	<0.0001	1.90 (1.82–1.97)	<0.0001	2.22 (1.90–2.59)	<0.0001
60–69	1.89 (1.84–1.95)	<0.0001	2.47 (2.32–2.62)	<0.0001	2.70 (2.25–3.23)	<0.0001
50–59	2.47 (2.37–2.57)	<0.0001	3.54 (3.15–3.97)	<0.0001	4.59 (3.55–5.92)	<0.0001
<50	3.37 (3.12–3.64)	<0.0001	3.26 (2.36–4.50)	<0.0001	5.51 (3.34–9.09)	<0.0001
Women						
≥80	1.36 (1.32–1.41)	<0.0001	1.34 (1.29–1.39)	<0.0001	1.95 (1.62–2.35)	<0.0001
70–79	1.56 (1.52–1.59)	<0.0001	1.68 (1.63–1.73)	<0.0001	1.77 (1.54–2.02)	<0.0001
60–69	1.58 (1.54–1.62)	<0.0001	2.06 (1.94–2.18)	<0.0001	2.21 (1.81–2.70)	<0.0001
50–59	1.86 (1.79–1.93)	<0.0001	2.91 (2.55–3.32)	<0.0001	3.72 (2.65–5.21)	<0.0001
<50	3.18 (2.90–3.49)	<0.0001	5.61 (3.75–8.40)	<0.0001	6.52 (3.09–13.75)	<0.0001

The analyses were adjusted for severe hypoglycemia. CI, confidence interval; HR, hazard ratio.

**Table 4:** Hazard ratios for all dementia types and subtypes stratified by age at diagnosis of type 2 diabetes mellitus in participants who underwent health checkups provided by the NHIS.

	All Dementia		Alzheimer Disease		Vascular Dementia	
	HR (95% CI)	*p* Value	HR (95% CI)	*p* Value	HR (95% CI)	*p* Value
≥80	1.45 (1.34–1.57)	<0.0001	1.34 (1.21–1.48)	<0.0001	1.98 (1.23–3.19)	<0.0001
70–79	1.68 (1.61–1.75)	<0.0001	1.77 (1.67–1.88)	<0.0001	1.96 (1.52–2.52)	<0.0001
60–69	1.77 (1.70–1.84)	<0.0001	2.33 (2.12–2.55)	<0.0001	3.00 (2.24–4.01)	<0.0001
50–59	2.11 (1.99–2.23)	<0.0001	3.16 (2.59–3.87)	<0.0001	4.02 (2.56–6.31)	<0.0001
<50	2.84 (2.50–3.23)	<0.0001	3.84 (2.14–6.90)	<0.0001	4.39 (1.75–11.01)	<0.0001

Analyses were adjusted for sex, smoking, drinking, physical activity, income, body mass index, systolic blood pressure, total cholesterol, triglyceride, fasting blood glucose, history of cardiovascular disease, and occurrence of severe hypoglycemia. CI, confidence interval; HR, hazard ratio; NHIS, National Health Insurance Service.

**Table 5:** Association between fasting glucose concentration and the risk of dementia subtypes in patients with type 2 diabetes who underwent health checkups provided by the NHIS.

Fasting Blood Glucose (mg/dL)	HR (95% CI)			*p* Value
All Dementia				
<95	0.91 (0.89–0.92)			<0.0001
95–124	1.00 (Ref)			
125–139	1.16 (1.13–1.19)			<0.0001
140–199	1.13 (1.10–1.16)			<0.0001
≥200	1.14 (1.09–1.18)			<0.0001
Alzheimer disease				
<95	0.89 (0.87–0.91)			<0.0001
95–124	1.00 (Ref)			
125–139	1.17 (1.12–1.22)			<0.0001
140–199	1.17 (1.12–1.22)			<0.0001
≥200	1.41 (1.32–1.52)			<0.0001
Vascular dementia				
<95	0.95 (0.86–1.04)			0.2436
95–124	1.00 (Ref)			
125–139	1.11 (0.93–1.32)			0.2528
140–199	1.29 (1.10–1.52)			0.0020
≥200	1.47 (1.15–1.89)			0.0024

Analyses were adjusted for age, sex, income, smoking, alcohol consumption, body mass index, diastolic and systolic blood pressures, low-density lipoprotein cholesterol, high-density lipoprotein cholesterol, and history of cardiovascular disease. CI, confidence interval; HR, hazard ratio; NHIS, National Health Insurance Service.

## Data Availability

The datasets presented in this article are not readily available because the data underlying this article were provided by the NHIS with permission. Requests to access the datasets should be directed to the corresponding author with permission from the NHIS.

## Supplementary Materials

The following supporting information can be downloaded at: https://www.mdpi.com/article/10.3390/jcm13154386/s1, Table S1: Diagnoses, procedures, and corresponding codes; Table S2: Baseline characteristics of the participants who underwent health checkups were grouped based on the development of dementia.
