# Supplementary material for: Association between Age at Diagnosis of Type 2 Diabetes and Subsequent Risk of Dementia and Its Major Subtypes
Source: J Clin Med. 2024 Jul 26;13(15):4386. doi: 10.3390/jcm13154386 (PMC11313191; doi:10.3390/jcm13154386)
Supplement: Supplementary file 1 [file jcm-13-04386-s001.zip › jcm-3063293-supplementary.pdf]

Table S1. Diagnoses, procedures, and corresponding codes

	ICD codes
Comorbidities	
Hypertension	I10, I15
Neoplasm	C00-97
History of CVD	
Myocardial infarction	I21, I22
CABG	O1640, O1641, O1642, O1643, O1644, O1645, O1646, O1647, O1648, O1649, OA640, OA641, OA642, OA647, OA648, OA649
PCI with stent	M6551, M6552, M6561, M6562, M6563, M6564, M6565, M6566, M6567
Unstable angina	I20.0
Angina pectoris	I20.1, I20.8. I20.9
Atrial fibrillation	I48
Heart failure	I50
Stroke	I60-I66
Hemorrhagic stroke	I60-I62
Ischemic stroke	I63-I64
Peripheral artery disease	ICD-10 code (I70-79) and procedure code (M6597, M6605, M6613, M6632, M6620, O0161–O0171, O1643-4, O1645~6), I70.2-3, I70.9, I73.1, I73.8-9
Severe hypoglycemia	E11.63, E12.63, E13.63, E14.63, E16.0, E16.1, E16.2 and hospitalization or a visit to an emergency department

CABG, coronary artery bypass graft; CVD, cardiovascular disease; ICD, International Classification of Diseases; PCI, percutaneous coronary intervention.

Table S2. Baseline characteristics of the participants who underwent health checkups according to the development of dementia.

	Dementia	No dementia	P
Total	102,244	1,133,172	
Age (years)	68.8±9.3	55.2±11.5	<.0001
Sex			<.0001
Male	41,065 (40.2)	707,593 (62.4)	
Female	61,179 (59.8)	425,579 (37.6)	
Smoking			<.0001
Never smoker	74,584 (72.9)	612,943 (54.1)	
Current smoker	13,963 (13.7)	217,254 (19.2)	
Ex-smoker	13,697 (13.4)	302,975 (26.7)	
Heavy drinking	16,466 (16.1)	321,864 (28.4)	<.0001
Body mass index (kg/m^2)	24.1±3.3	24.3±3.3	<.0001
Waist circumference (cm)	82.9±8.6	82.6±9.0	<.0001
Regular exercise	27,903 (27.3)	386,230 (34.1)	<.0001
Income			<.0001
Q1	43,830 (42.9)	477,363 (42.1)	
Q2	16,169 (15.8)	211,480 (18.7)	
Q3	18,054 (17.7)	208,345 (18.4)	
Q4	24,191 (23.7)	235,984 (20.8)	
Systolic blood pressure (mmHg)	128.7±16.1	125.0±15.4	<.0001
Diastolic blood pressure (mmHg)	78.1±10.2	77.9±10.3	<.0001
Total cholesterol (mg/dl)	202.2±42.3	202.6±40.4	0.0059
Triglyceride (mg/dl)	114.3±93.9	150.9±118.3	<.0001
HDL-C (mg/dl)	53.9±30.1	53.9±24.9	0.3322
LDL-C (mg/dl)	120.6±47.6	120.2±54.3	0.0115
Fasting glucose (mg/dl)	107.9±34.0	110.4±42.1	<.0001
Comorbidities			
Type 2 diabetes mellitus	39,438 (38.6)	327071 (28.9)	<.0001
Hypertension	53,857 (52.7)	322650 (28.5)	<.0001
Dyslipidemia	34,343 (33.6)	252329 (22.3)	<.0001
Cancer	5,111 (5.0)	43081 (3.8)	<.0001
Chronic kidney disease	583 (0.6)	3605 (0.3)	<.0001
History of CVD	13,499 (13.2)	51151 (4.5)	<.0001

Values are expressed as mean ± standard deviation or number (%).
CVD, cardiovascular disease; HDL-C, high-density lipoprotein cholesterol; LDL-C, low-density lipoprotein cholesterol; NHIS, National Health Insurance Service; Q, quartile.
